# Laminin α5_CD239_Spectrin is a candidate association that compensates the linkage between the basement membrane and cytoskeleton in skeletal muscle fibers

**DOI:** 10.1016/j.mbplus.2022.100118

**Published:** 2022-08-06

**Authors:** Yamato Kikkawa, Masumi Matsunuma, Ryuji Kan, Yuji Yamada, Keisuke Hamada, Motoyoshi Nomizu, Yoichi Negishi, Shushi Nagamori, Tatsushi Toda, Minoru Tanaka, Motoi Kanagawa

**Affiliations:** a Department of Clinical Biochemistry, Tokyo University of Pharmacy and Life Sciences, Tokyo 192-0392, Japan; b Department of Drug Delivery and Molecular Biopharmaceutics, Tokyo University of Pharmacy and Life Sciences, Tokyo 192-0392, Japan; c Department of Laboratory Medicine, The Jikei University School of Medicine, 105-8461 Tokyo, Japan; d Department of Neurology, Graduate School of Medicine, The University of Tokyo, 113-0033 Tokyo, Japan; e Department of Regenerative Medicine, Research Institute, National Center for Global Health and Medicine, Tokyo, Japan; f Division of Molecular Brain Science, Kobe University Graduate School of Medicine, Kobe, Hyogo 650-0017, Japan; g Department of Cell Biology and Molecular Medicine, Ehime University Graduate School of Medicine, Toon, Ehime 791-0295, Japan

**Keywords:** Basement membrane, Laminin, CD239, BCAM, Spectrin, Muscular dystrophy

## Abstract

The linkage between the basement membrane (BM) and cytoskeleton is crucial for muscle fiber stability and signal transduction. Mutations in the linkage molecules can cause various types of muscular dystrophies. The different severities and times of onset suggest that compensatory linkages occur at the sarcolemma. Cluster of differentiation 239 (CD239) binds to the α5 subunit of laminin-511 extracellularly and is connected to spectrin intracellularly, resulting in a linkage between the BM and cytoskeleton. In this study, we explored the linkage of laminin α5_CD239_spectrin in skeletal muscles. Although laminin α5, CD239, and spectrin were present in embryonic skeletal muscles, they disappeared in adult skeletal muscle tissues, except for the soleus and diaphragm. Laminin α5_CD239_spectrin was localized in the skeletal muscle tissues of Duchenne muscular dystrophy and congenital muscular dystrophy mouse models. The experimental regeneration of skeletal muscle increased the CD239-mediated linkage, indicating that it responds to regeneration, but not to genetic influence. Furthermore, *in silico* analysis showed that laminin α5_CD239_spectrin was upregulated by steroid therapy for muscular dystrophy. Therefore, CD239-mediated linkage may serve as a therapeutic target to prevent the progression of muscular dystrophy.

## Highlights

• Laminin α5_CD239_spectrin complex is a candidate linkage in sarcolemma.
• The linkage molecules are expressed in embryonic and regenerative muscle fibers.
• CD239 expression is upregulated by steroid therapy for muscular dystrophy.
• The compensatory linkage may be a therapeutic target for muscular dystrophy.

## Introduction

Each skeletal muscle fiber is surrounded by a sheet-like extracellular matrix, called the basement membrane (BM). BM serves as a scaffold to organize muscular development and orient its regeneration. Binding of the muscle plasma membrane, called the sarcolemma, to the BM is crucial for muscle fiber stability and signal transduction. Mutations in multiple BM proteins, cell surface receptors, transmembrane accessory proteins, cytoskeletal proteins, and glycosylating enzymes cause muscular dystrophies of different severities and varying times of onset [1]. Like all BMs, skeletal muscle fiber BM contains four major matrix proteins: laminins, type IV collagens, nidogens, and heparan sulfate proteoglycans [2]. Laminins are large glycoproteins composed of α, β, and γ chains [3]. So far, 5 α-, 3 β-, and 3 γ-chains have been characterized, and 19 different laminin heterotrimeric isoforms have been identified in various tissues and cell culture media. The major laminin isoform in skeletal muscle BM is laminin-211 (LM-211: α2, β1, γ1) [4]. Mutations in *LAMA2* encoding laminin α2 cause muscular dystrophy congenital type 1A [5]. Cell adhesion to laminins is mediated by various receptors, including integrins, syndecans, dystroglycan, and CD239 [3]. Cell-surface receptors mainly bind to the laminin-type globular (LG) domain at the C-terminus of α chain. LG domain of the α2 chain binds to receptors on the surface of myofibers, including dystroglycan (DG) and integrin α7β1 [5]. DG forms a complex with dystrophin and its associated glycoproteins, while integrin α7β1 is associated with cytoplasmic adaptor proteins. Both cellular apparatuses play pivotal roles in the anchoring of muscle cytoskeleton to the BM. Mutations in anchoring molecules cause various types of muscular dystrophy [6]. DG consists of α and β subunits, and the mannosyl-*O*–linked carbohydrate of the α subunit is involved in the binding to laminin α2 [7]. Furthermore, mutations that affect the enzymes responsible for glycosylation of α-DG cause dystroglycanopathies, which manifest as muscular dystrophies [8]. Therefore, the functional recovery of anchoring apparatuses is a potential therapeutic strategy for muscular dystrophy.

**Fig. 1:**
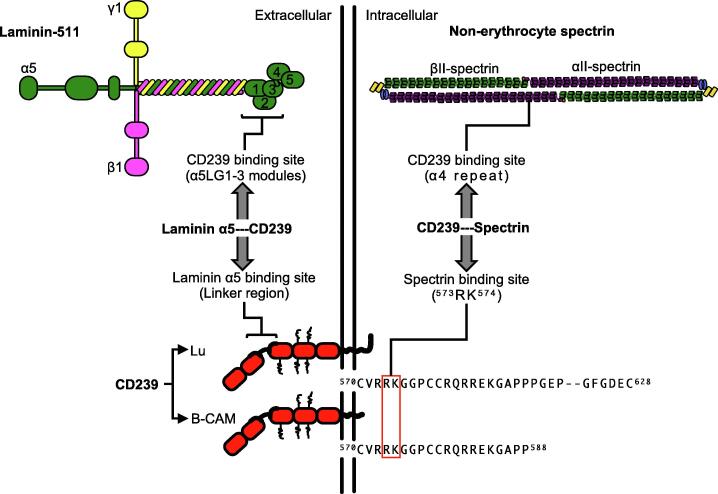
Shema of CD239-mediated linkage. CD239 linker region binds to LG1–3 modules of laminin α5 extracellularly [30, 31]. CD239 has two isoforms, Lu and B-CAM with cytoplasmic tails of different lengths. The common region of the Lu and B-CAM cytoplasmic tails contains a spectrin-binding motif, R^573^K^574^ [11, 14]. The motif connects to α4 repeat of αII-spectrin intracellularly [14]. CD239 associates with laminin α5 and spectrin, resulting in the linkage between basement membrane and cytoskeleton.

Among laminin receptors, basal cell adhesion molecule (B-CAM), also known as the Lutheran blood group glycoprotein (Lu), is an immunoglobulin superfamily transmembrane protein [9]. Lu and B-CAM are classified as cluster of differentiation 239 (CD239) molecules [10]. Lu and B-CAM have the same extracellular domain, containing one V-set, one C1-set, and three I-set domains (V-C1-I-I-I), with cytoplasmic tails of different lengths. B-CAM lacks the COOH-terminal and 40 amino acids of the Lu cytoplasmic tail. The common region of the Lu and B-CAM cytoplasmic tails contains a spectrin-binding motif [11]. Hereafter, because we focus on the binding of spectrin to the common region of Lu and B-CAM cytoplasmic tails, they will be referred to as CD239. The spectrin-binding motif of CD239 intracellularly binds to the erythrocyte and non-erythrocyte spectrins [11], [12], [13], [14]. CD239 is also a specific receptor for laminin α5, a major extracellular component of BM [15], [16]. Therefore, CD239 forms a linkage with laminin α5 and spectrin in the plasma membrane (Fig. 1). Although CD239-mediated linkage impacts the cell adhesion of erythrocytes, it is unclear whether the linkage plays a role in various tissues, including skeletal muscle tissues.

In this study, we first observed the co-distribution of laminin α5, CD239, and spectrin in embryonic skeletal muscle fibers. They disappeared in adult skeletal muscle tissues, except in the soleus and diaphragm. The expression levels of linkage molecules were upregulated in the skeletal muscles of Duchenne muscular dystrophy (DMD) and congenital muscular dystrophy (CMD) mouse models. Their induced expression appeared to be due to the regeneration of the skeletal muscle. *In silico* analysis showed that their expression in skeletal muscle tissues were induced by steroids that were administered during therapy. Hence, induction of CD239-mediated linkage may be a therapeutic strategy for muscular dystrophy.

## Results

### Linkage between the BM and cytoskeleton in fetal and adult mouse skeletal muscles

**Fig. 2:**
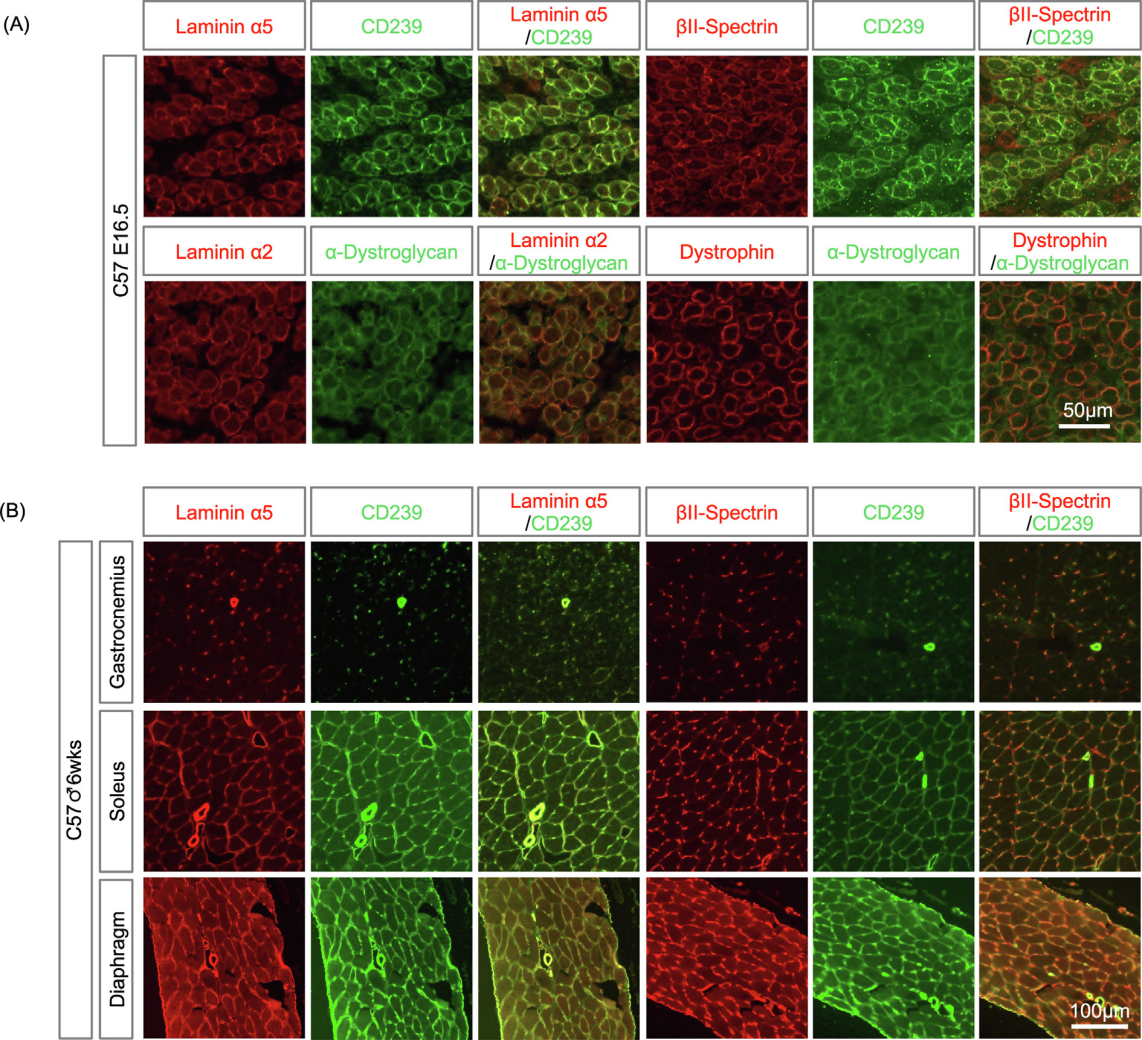
Expression levels of laminin α5, cluster of differentiation 239 (CD239), and βII-spectrin in fetal and adult mouse skeletal muscles. (A) Skeletal muscle tissues of E16.5 embryo. Frozen tissue sections were stained with antibodies against laminin α5, CD239, βII-spectrin, laminin α2, α-dystroglycan, and dystrophin, as indicated in panels. Bars: 50 μm. (B) Skeletal muscle tissues of 6-week-old male mouse. Gastrocnemius (upper panel), soleus (middle panel), and diaphragm (lower panel). Tissue sections were stained with antibodies as indicated in the panels. Bar: 100 μm.

**Fig. 3:**
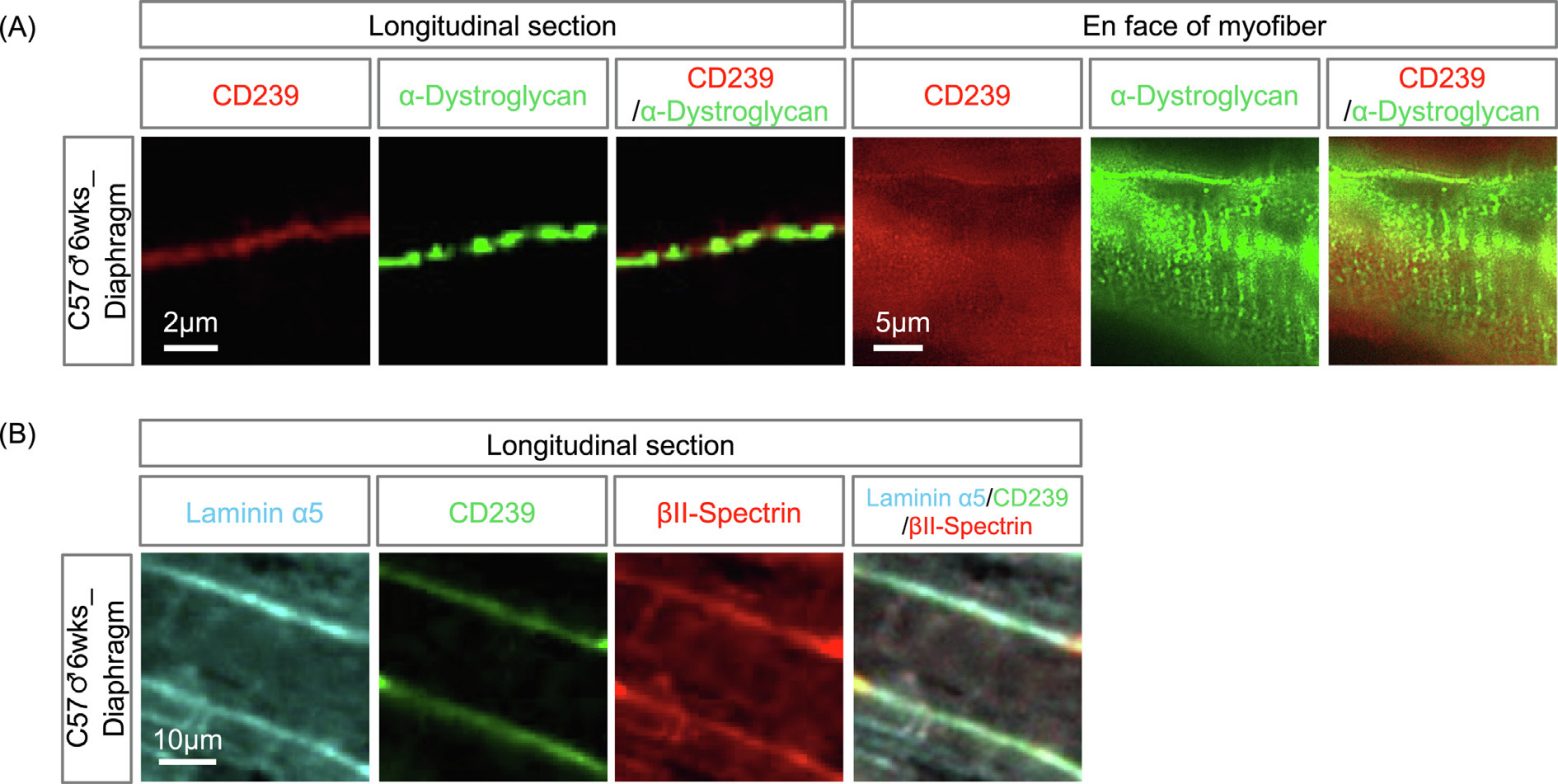
Localization of laminin α5, CD239, and βII-spectrin in sarcolemma. (A) Longitudinal (left panel) and en face (right panel) views of myofiber. Longitudinal sections of diaphragm were stained with antibodies against CD239 and α-dystroglycan. Thick cryosection was used for en face view. Bars: 2 and 5 μm in left and right panels, respectively. (B) Triple immunostaining of the linkage components. Longitudinal section of diaphragm was stained with antibodies against laminin α5, CD239 and βII-spectrin. Bar: 10 μm.

The α5-containing laminins are localized in the BM of embryonic skeletal muscles [17], [18]. CD239 is expressed on the surface of embryonic muscle fibers [19]. We first examined the colocalization of laminin α5, CD239, and spectrin in the skeletal muscles of E16.5 mouse embryos. The intracellular domain of CD239 binds to the αII-spectrin in non-erythrocytes (Fig. 1). Unfortunately, no antibody recognizing mouse αII-spectrin is available for immunohistochemistry. As βΙΙ-spectrin usually forms antiparallel dimers with the αII subunit, we used an anti-βΙΙ-spectrin polyclonal antibody that is applicable for immunostaining in mouse tissues in this study. As shown in previous studies, CD239 was expressed on the surface of embryonic muscle fibers, and laminin α5 was present in the myofiber BM (Fig.  2A). The staining of βΙΙ-spectrin was co-localized with CD239 in the plasma membrane of embryonic muscle fibers, suggesting that the CD239-mediated linkage is in the embryonic sarcolemma. However, the linkage disappeared in the muscle fibers of the adult skeletal muscle tissues, except for the soleus and diaphragm (Fig. 2B). In contrast, a major linkage (DG-mediated linkage), including laminin α2, DG, and dystrophin, was constitutively localized in the sarcolemma of fetal and adult skeletal muscles (Fig.  2A and S1). Laminin α5_CD239_spectrin was localized with laminin α2, DG, and dystrophin in the soleus and diaphragm, similar to the embryonic skeletal muscles (Fig.  2 and S1). They also remained in the blood vessels of adult skeletal muscles. DG-mediated linkage is localized at adjacent to costameres of skeletal muscle [20]. We observed the double staining of CD239 and DG on longitudinal sections and en face of a diaphragm myofiber at higher magnification (Fig. 3A). Although the staining of DG exhibited the costameric pattern, CD239 was localized in entire sarcolemma. Furthermore, longitudinal section of diaphragm was triply stained with antibodies to laminin α5, CD239 and βII-spectrin (Fig.  3B). The staining of CD239 was overlapped with those of laminin α5 and βII-spectrin, suggesting that CD239-mediated linkage was formed at sarcolemma.

### CD239-mediated linkage in skeletal muscles of DMD and CMD model mice

**Fig. 4:**
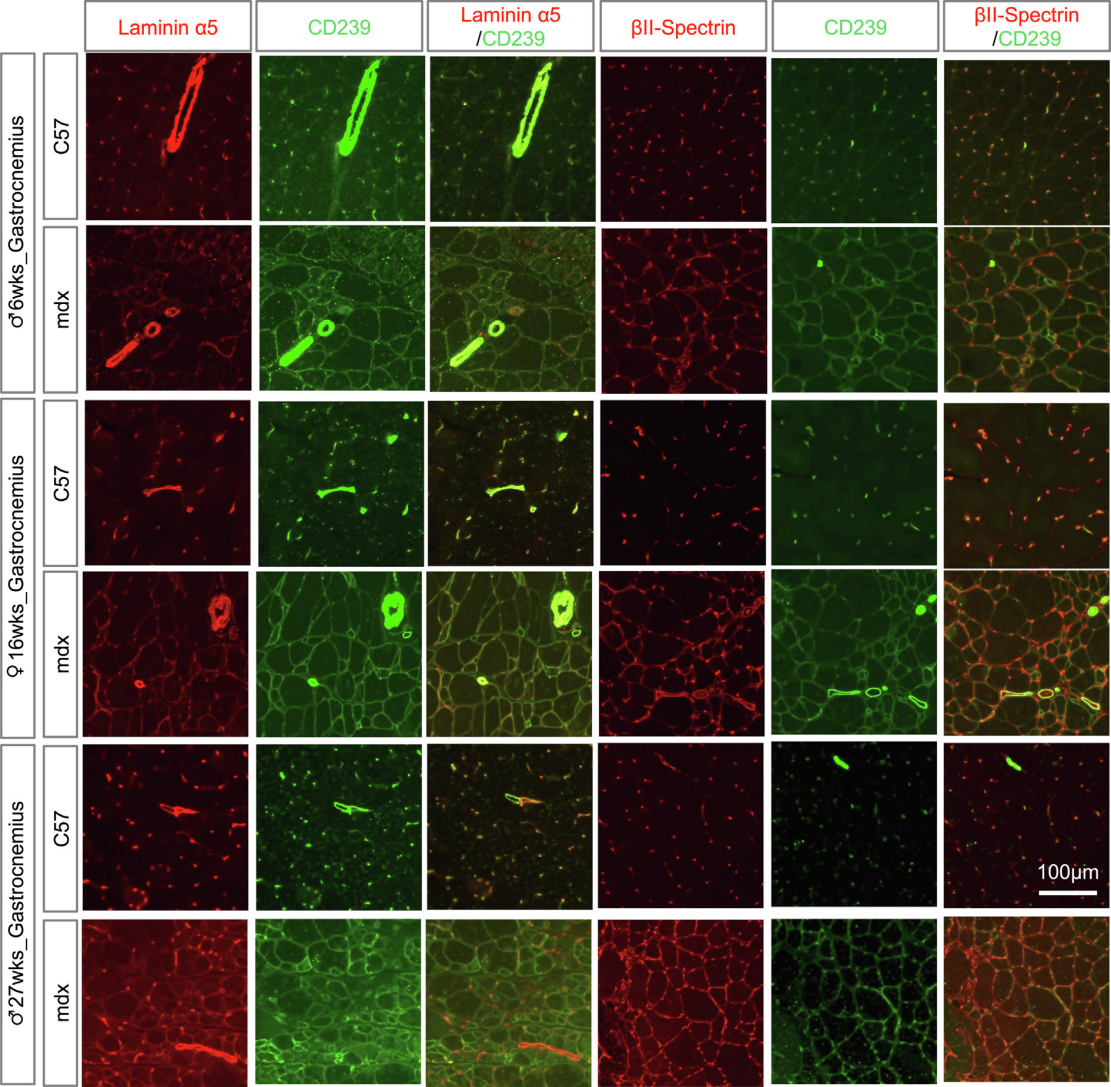
Expression levels of laminin α5, CD239, and βII-spectrin in skeletal muscles of Duchenne muscular dystrophy (DMD) model mice. Gastrocnemius tissue sections of the control and mdx (6- and 27-week-old male and 16-week-old female) mice were stained with antibodies against laminin α5, CD239, and βII-spectrin, as indicated in the panels. Bar: 100 μm.

Expression levels of laminin α5 are elevated in the muscles of many dystrophic patients [21]. Laminin α5 expression levels are also upregulated in the dystrophin (mdx) mutant, a model of DMD. Previous studies led us to hypothesize that CD239 and βΙΙ-spectrin were co-localized with laminin α5 in myofibers of muscular dystrophic mice. In this study, we performed immunohistochemistry on tissues of 6-, 16-, and 27-week-old mdx mice (Fig. 4). In the gastrocnemius of 6- and 16-week-old mdx mice, the expression levels of CD239 and βΙΙ-spectrin were readily observed in collapsed myofibers, but laminin α5 expression was weak. Laminin α5 was expressed in regenerative myofibers with centrally located nuclei in 27-week-old mdx mice. Although laminin α2 expression levels were maintained in the muscle fibers lacking dystrophin, DG expression levels were reduced at the cell surface of myofibers (Fig. S2). Laminin α5_CD239_spectrin appeared in mdx mice with severe symptoms.

**Fig. 5:**
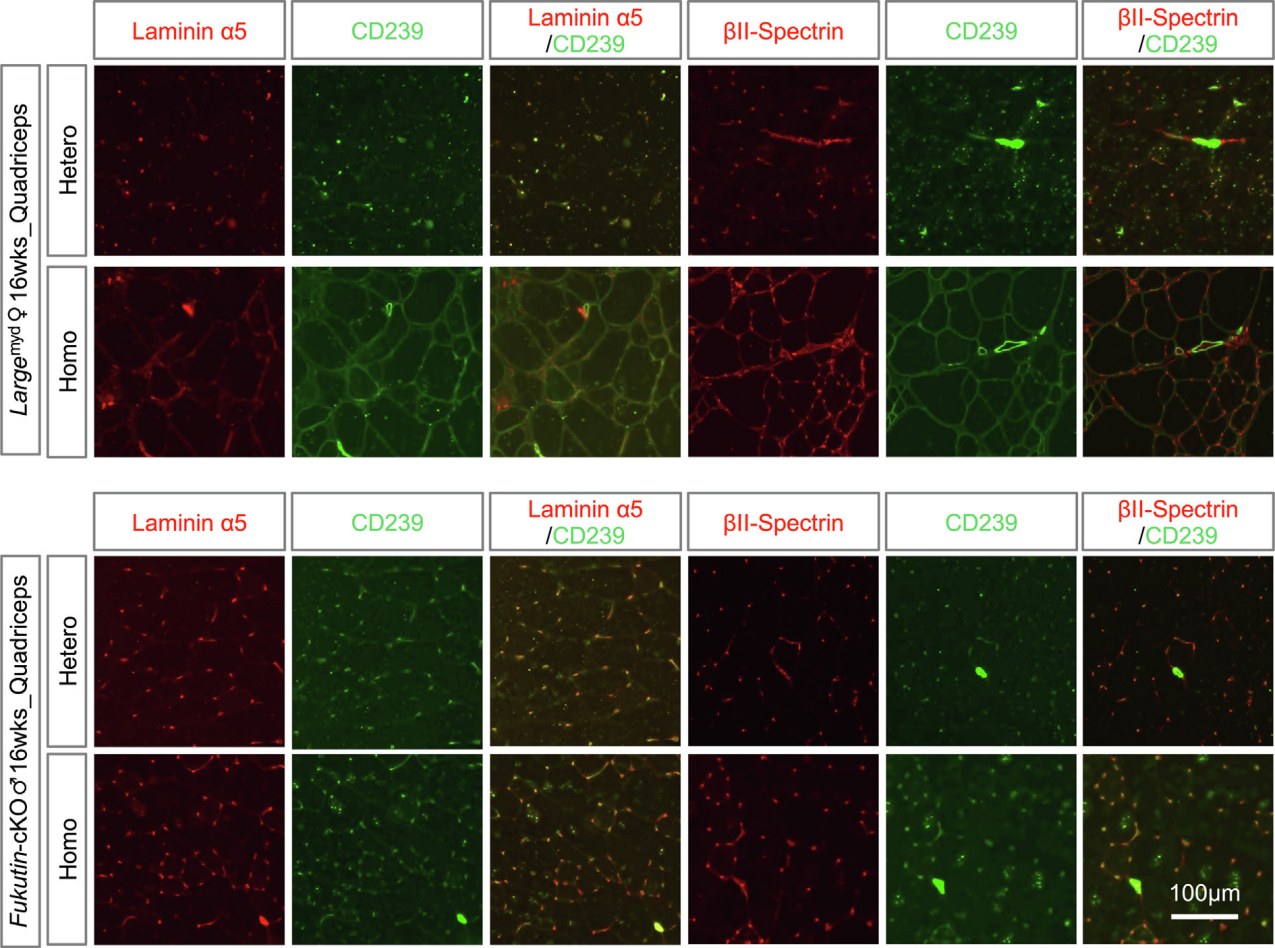
Expression levels of laminin α5, CD239, and βII-spectrin in skeletal muscles of congenital muscular dystrophy (CMD) model mice. (A) Quadriceps muscles of heterozygous and homozygous *Large*^myd/myd^ mice (16-week-old female). The tissue sections were stained with series of antibodies indicated in the panels. (B) Quadriceps muscles of heterozygous and homozygous MCK*Fukutin*-cKO mice (16-week-old female). Bar: 100 μm.

DG binds to laminin α2 via the mannosyl-*O*–linked carbohydrate of the α subunit [7]. Mutations in glycosylating enzymes, such as Fukutin and Large, cause muscular dystrophies of different severities and times of onset [22]. In this study, we performed immunohistochemistry on quadriceps tissues of 16-week-old *Large*^myd/myd^ and MCK-*Fukutin*-conditional knockout mice (cKO) (Fig 5 and S3). MCK-*Fukutin*-cKO mice exhibit milder phenotype of muscular dystrophy than mdx and *Large*^myd/myd^ mice [23], [24]. In the quadriceps of *Large*^myd/myd^ mice, the expression levels of CD239 and βΙΙ-spectrin were observed in the myofibers, but laminin α5 expression was weak. In the skeletal muscle of MCK-*Fukutin*-cKO mice, laminin α5_CD239_spectrin levels were negligible. The appearance of CD239-mediated linkage seems to be associated with disease severity and response to regeneration during disease progression.

Laminin α1, α3 and α4 were not expressed in adult and dystrophic myofibers (Fig. S2 and S3). Laminin α4 was exclusively observed in blood vessels of skeletal muscle. The results suggested that laminin α5 was a compensative component in regenerative myofibers.

### Induction of laminin α5_CD239_spectrin in regenerating skeletal muscles

**Fig. 6:**
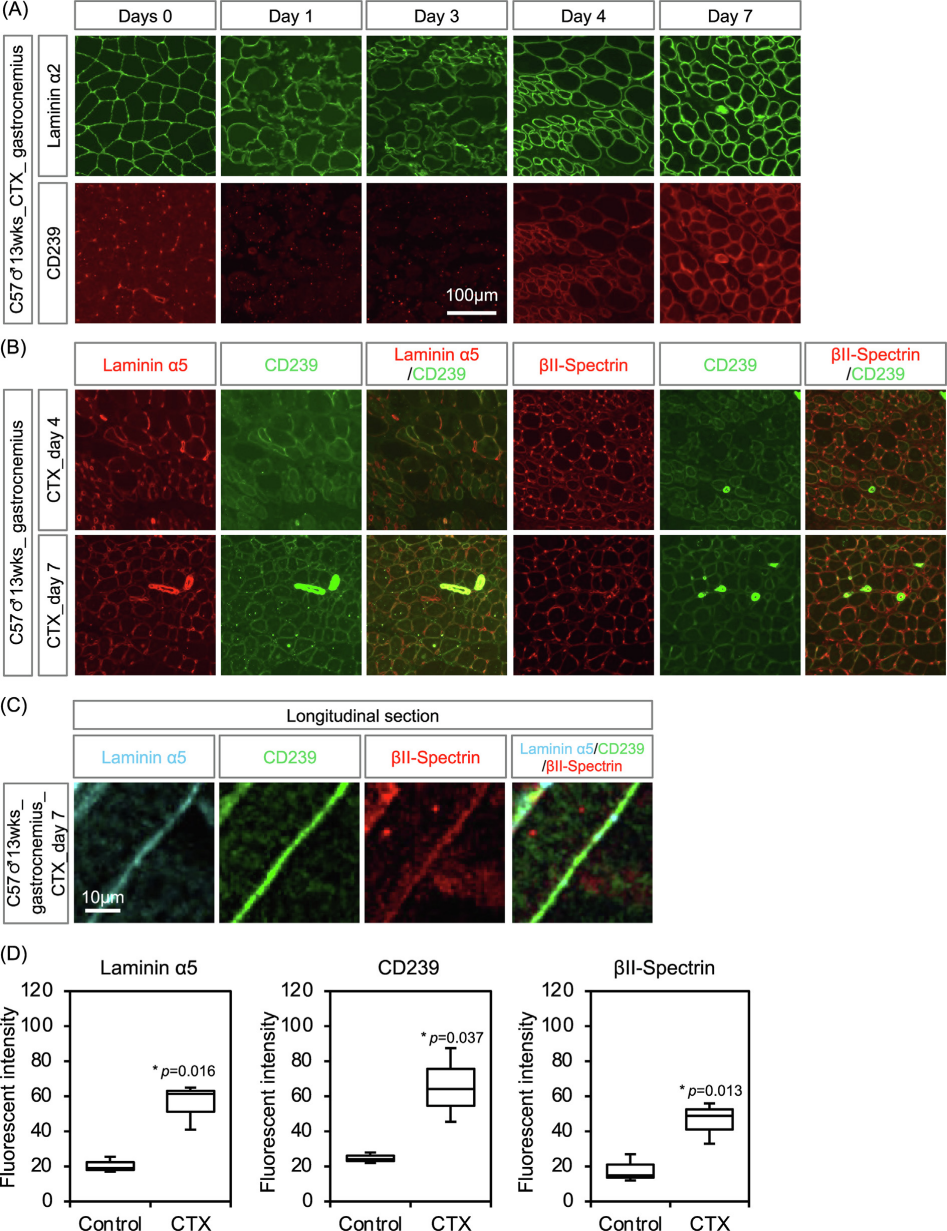
Expression levels of CD239 in regenerative skeletal muscles. (A) Time course of CD239 expression levels in regenerative skeletal muscles. The frozen sections of day 0, 1, 3, 4, and 7 after cardiotoxin (CTX)-induced regenerative gastrocnemius muscles were doubly stained with antibodies against laminin α2 (upper panel, green) and CD239 (lower panel, red). (B) Linkage molecules of sarcolemma in regenerative skeletal muscles. The frozen sections of day 4 and 7 after CTX-induced regenerative gastrocnemius muscles were stained with antibodies against laminin α5, CD239, βII-spectrin, as indicated in the panels. Bar: 100 μm. (C) Triple immunostaining on longitudinal section of CTX-induced regenerative gastrocnemius muscles. Bar: 10 μm. (D) Quantitative analysis of CD239-mediated linkage. Fluorescence intensities of laminin α5, CD239, and βII-spectrin were measured at surrounding regions of day 7 gastrocnemius muscle fibers treated with saline or CTX. Box-and-whisker plots show the median, 25th and 75th percentiles, and minimum and maximum values (n = 3 mice/group). Statistical significance was determined using Welch’s *t* test. *p < 0.05.

**Fig. 7:**
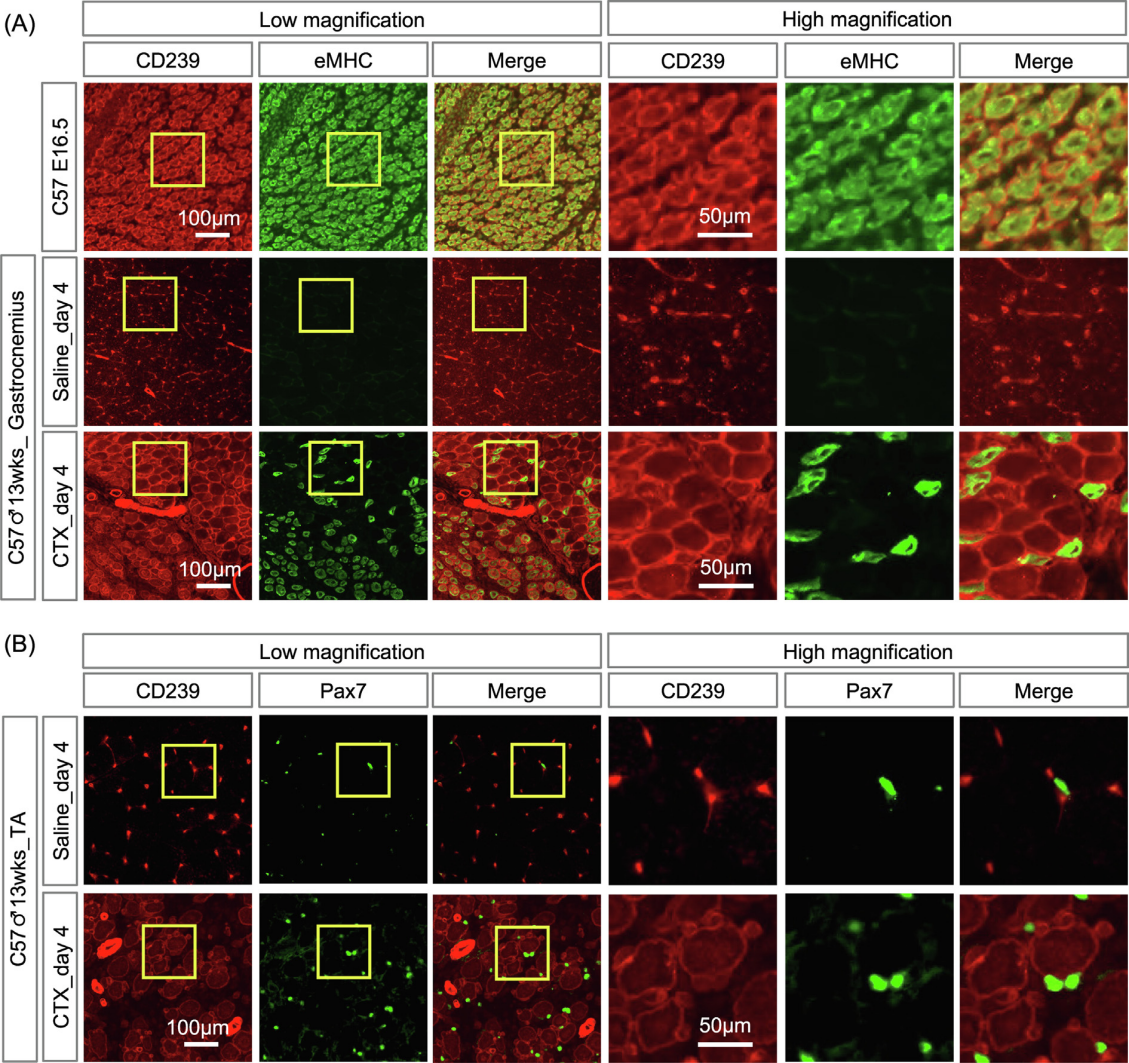
Expression levels of CD239 in regenerating muscle fibers and muscle progenitor cells. (A) CD239 expression levels in embryonic myosin heavy chain (eMHC)-positive-muscle cell fibers. The frozen sections were prepared from E16.5 embryos (upper panel) and day 4 gastrocnemius muscles treated with saline (middle panel) or CTX (lower panel). They were doubly stained with antibodies against CD239 (red) and eMHC (green). eMHC is a marker of immature muscle cell fibers. (B) Expression levels of CD239 in satellite cells. The frozen sections of day 4 after CTX-induced regenerative tibialis anterior (TA) muscles were doubly stained with antibodies against CD239 (red) and paired box 7 (Pax7) (green). Pax7 is a marker for satellite cells. Bars: 100 and 50 μm in left and right panels, respectively.

During muscle regeneration, some genes expressed in embryonic myotubes are re-expressed [25]. To examine whether regeneration leads to the appearance of CD239-mediated linkage, we examined the temporal expression patterns of CD239 after cardiotoxin (CTX)-induced injury of gastrocnemius muscles. The expression of CD239 was induced on day 4 and was maintained on day 7 after CTX-induced injury (Fig. 6A). Myofibers in the regenerating skeletal muscles were visualized by staining with laminin α2. Laminin α5 and βΙΙ-spectrin were also co-localized with CD239 in regenerating myotubes with centrally located nuclei (Fig. 6B). Furthermore, longitudinal section of regenerating myotube was triply stained with antibodies to laminin α5, CD239 and βII-spectrin (Fig. 6C). The staining of CD239 was overlapped with those of laminin α5 and βII-spectrin, suggesting that CD239-mediated linkage was formed at regenerative sarcolemma. The results also indicated that the muscular regeneration led to the expression of linkage molecules. Quantitative analysis showed that the fluorescence intensities of laminin α5 (*p* = 0.016), CD239 (*p* = 0.037), and βΙΙ-spectrin (*p* = 0.013) were significantly increased in regenerating myofibers (Fig. 6D). Furthermore, we stained sections of regenerating muscles for the embryonic isoform of myosin heavy chain (eMHC) to label the region of active construction. In embryonic skeletal muscles, all myofibers were positive for eMHC and stained with the anti-CD239 antibody (Fig. 7A). In regenerating skeletal muscles, the staining of CD239 was observed not only in eMHC-positive myofibers, but also in growing eMHC-negative myotubes. Satellite cells (SCs) serve as skeletal muscle-specific stem cells for muscle regeneration after injury [26]. Therefore, SCs were visualized by staining for paired box 7 (Pax7), a transcriptional regulator of myogenesis. CD239 expression in SCs was negligible in the Pax7-positive cells (Fig. 7B).

### CD239-mediated linkage in steroid therapy of muscular dystrophy

**Fig. 8:**
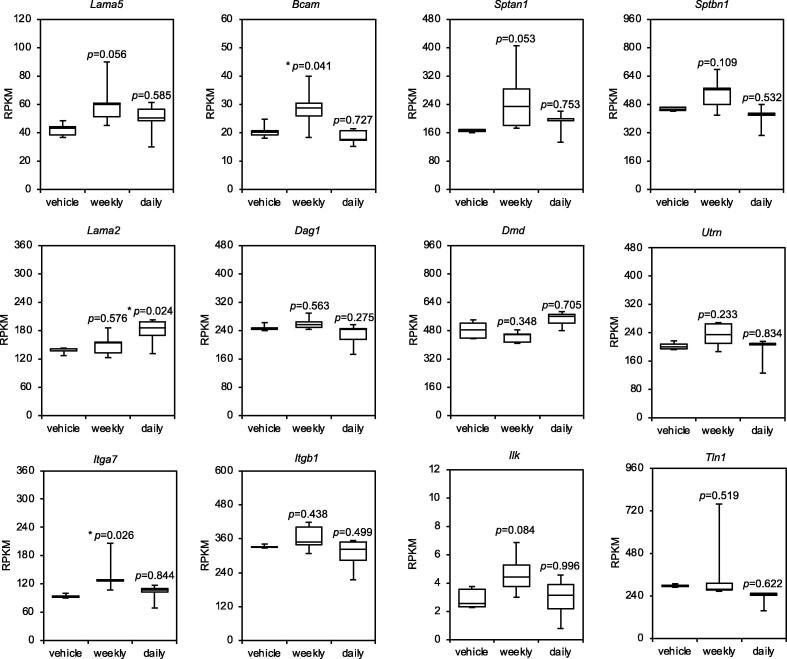
Expression levels of laminin α5_CD239_spectrin in steroid-treated murine dystrophic muscles. For *in silico* analysis, the RNA-seq dataset was obtained from the Gene Expression Omnibus (GEO) database (GSE95682). The transcriptional profile was prepared from the quadriceps muscles of 6-month-old mdx mice treated with or without prednisone (weekly and daily) (n = 5 mice/group). Steroids were administered to mdx mice daily or weekly for four weeks. The counts of reads per kilobase of transcript per million mapped reads (RPKM) were extracted from the dataset. Box-and-whisker plots show the median, 25th and 75th percentiles, and minimum and maximum values (n = 5 mice/group). Data were analyzed using one-way analysis of variance (ANOVA) with Dunnett’s post-hoc test. *P < 0.05. Lama5, laminin subunit α5; Bcam, basal cell adhesion molecule; CD239, cluster of differentiation 239; Sptan1, αII-spectrin; Sptbn1, βII-spectrin; Lama2, laminin subunit α2; Dag1, dystroglycan 1; Dmd, Duchenne muscular dystrophy; Itga7, integrin α7; Itgb1, integrin β1; Ilk, integrin-linked protein kinase; Tln1, talin1.

Glucocorticoid (GC) steroids, such as deflazacort and prednisolone, are used to treat DMD. In mdx mice, deflazacort improves the muscle regeneration and growth after injury [27], [28]. An 8-week study of prednisolone in mdx mice showed improved specific force and decreased number of centrally nucleated myofibers [29]. However, despite the beneficial effects of steroid treatment in muscular dystrophy, the underlying mechanisms remain unclear. These findings allowed us to hypothesize that laminin α5_CD239_spectrin induced by GC steroid treatment was involved in the improvement of DMD symptoms. Recently, Quattrocelli et al. showed a profile of gene expression patterns in skeletal muscles of mdx mice treated with a vehicle and prednisone (weekly and daily) [30]. To investigate the CD239-mediated linkage, we utilized a dataset from the Gene Expression Omnibus (GEO) database. The gene expression involved in the linkage of the sarcolemma was visualized using a heatmap (Fig. S4). The gene expression levels of laminin α5, CD239, and αII/βΙΙ-spectrins seemed to be upregulated in the skeletal muscles of mdx mice treated with weekly dose of prednisone, which had positive effects on muscular dystrophy compared to the mice that received a daily dose of prednisone. Furthermore, we extracted RNA count data and evaluated them statistically (Fig. 8). The gene expression levels of CD239 (*p* = 0.041) and integrin α7 (*p* = 0.026) were significantly upregulated with weekly dosing of prednisone. The gene expression levels of laminin α5 (*p* = 0.056), αII-spectrin (*p* = 0.053), βΙΙ-spectrin (*p* = 0.109) and integrin-linked protein kinase (*p* = 0.084) in the skeletal muscles treated with weekly dosing of prednisone tended to increase in comparison with the other genes. The expression levels of other genes in the weekly treated mice were comparable to those in control mice. *In silico* analysis revealed that laminin α5_CD239_spectrin is a candidate complex that can compensate for the weakened adhesion of myofibers to the BM in muscular dystrophy.

## Discussion

**Fig. 9:**
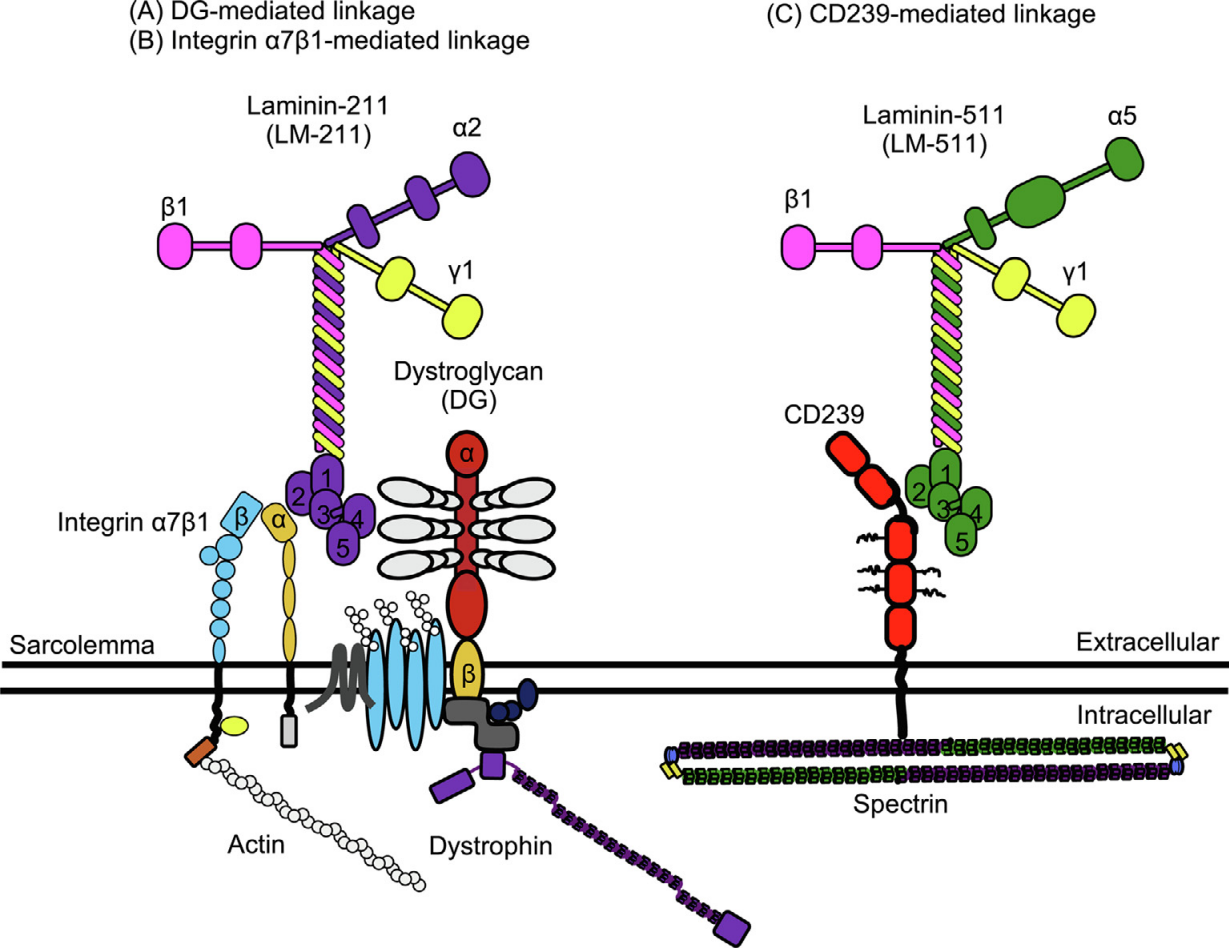
Schema of the linkage between the basement membrane (BM) and cytoskeleton in skeletal muscles. (A) Dystrophin-associated glycoprotein complex (DGC). The membrane-associated DGC is a major linkage of sarcolemma. Dystroglycan (DG) binds to the α2 subunit of laminin-211 (LM-221) extracellularly and to dystrophin intracellularly. (B) Integrin-mediated cell adhesion in muscle fibers. Integrin α7β1 binds to LG1–3 modules of laminin α2 extracellularly and connects to actin-polymer intracellularly. (C) Laminin α5_CD239_spectrin in regenerative muscle fibers. CD239 binds to laminin α5 extracellularly and connects to spectrin intracellularly.

To withstand the mechanical force generated during contraction, skeletal muscle fibers have specialized linkages between the BM and cytoskeleton. Firm linkages in the sarcolemma are mediated via DG and integrin α7β1 (Fig. 9). Mutations in linkage molecules in the sarcolemma cause muscular dystrophies with different severities and times of onset, indicating that the various types of muscular dystrophies may be due to the compensation of linkages. In this study, we discovered that laminin α5_CD239_spectrin is a candidate association that compensates the linkage between the BM and cytoskeleton in skeletal muscle fibers (Fig. 9).

CD239 binds extracellularly to the α5 subunit of laminin-511 and is intracellularly connected to spectrin. Currently, the only known extracellular ligand of CD239 is laminin α5. On the other hand, laminin α5 can bind to other receptors such as the integrin α7β1 complex, which is a major integrin in skeletal muscle. Non-erythrocyte spectrin can also interact with the other cytoplasmic proteins such as ankyrin. Therefore, CD239 seems to be a key molecule for the formation of the compensatory linkage in regenerative myofibers. Because the staining of CD239 were mostly merged with laminin α5 and βII-spectrin in the plasma membrane of muscle fibers, it is likely that laminin α5, CD239, and spectrin are complexed at sarcolemma considering published known interactions [11], [14], [31], [32]. CD239, also known as Lu or B-CAM, is an Ig superfamily transmembrane protein. CD239 (Lu) was initially studied in red blood cells (RBCs) [33]. Kroviarski et al. reported that Lu and B-CAM directly interact with erythrocyte αI-spectrin in the cytoplasm [11]. Furthermore, the interaction of spectrin with Lu affects the adhesion of RBCs to laminin α5 [12], [13]. Lu and B-CAM also interact with non-erythrocyte αII-spectrin and modulate the adhesion of epithelial cells to laminin α5 [14]. Because the disruption of Lu and B-CAM interactions with spectrin enhances cell adhesion to laminin α5, the physiological strength of CD239-mediated linkage might not be as potent. It is important to show biochemical evidence of laminin α5-CD239-spectrin complex; however, it has not been obtained possibly because detergent extraction may disrupt their interactions. Although the issue should be addressed in the near future, this linkage may be helpful in preventing the progression of muscular dystrophy.

Although laminin α5_CD239_spectrin is observed in embryonic skeletal muscles, it disappears in adult skeletal muscles, except for the soleus muscle and diaphragm. The transition of the linkage components allowed us to hypothesize that it plays a role in muscular development and regeneration. However, CD239-deficient mice were born at the Mendelian ratio, developed normally, and exhibited no defects in physiological conditions [34]. The CD239-mediated linkage seems to disappear as a non-essential component of the skeletal muscle, except for the soleus muscle and diaphragm. Although it is difficult to evaluate the significance of the linkage in skeletal muscle, it may be involved in the growth of myofibers rather than muscular development and regeneration. Mdx mice exhibit progressive degeneration in the diaphragm rather than that in the limb muscles [35], which indicates that the diaphragm is susceptible to stressful stimuli, such as contraction. Therefore, myofibers of the diaphragm may always suffer from mechanical stress to induce laminin α5_CD239_spectrin. The gene expression profile of the soleus muscle is similar to that of the diaphragm, indicating that both tissues are distinct from other limb skeletal muscles [36]. The human soleus muscle specifically plays an important role in maintaining the standing posture and is responsible for pumping venous blood back into the heart from the periphery. Similar to the diaphragm, myofibers of the mouse soleus muscle may also suffer from mechanical stress.

Our results showed that laminin α5_CD239_spectrin appears in regenerative myofibers in skeletal muscles after CTX-induced injury. They are readily observed in collapsed myofibers of mdx mice with severe symptoms. Although the laminin α5_CD239_spectrin expression was low, if any, in the skeletal muscles of 16-week-old MCK-*Fukutin*-cKO mice, the linkage was observed in the myofibers of same-aged *Large*^myd/myd^ mice. Because *Large*^myd/myd^ mice exhibit more severe dystrophy than MCK-*Fukutin*-cKO mice [23], [24], regenerating myofibers are readily observed in the skeletal muscles of *Large*^myd/myd^ mice. The expression of laminin α5_CD239_spectrin strongly reflects regeneration rather than the genetic influence. Muscular dystrophy congenital type 1A (MDC1A) is caused by mutations in *LAMA2* encoding laminin α2 [5]. Patton et al showed that laminin α5 is upregulated in skeletal muscle of *Lama2*-/- mice and patients [21]. Furthermore, CD239 expression is upregulated in adult myofibers of laminin α5 transgenic mice [19]. Although spectrin expression is uncertain, it is likely that CD239-mediated linkage is formed in myofiber lacking laminin α2. Following injury, activated SCs lead to the formation of new myofibers via myoblast fusion [37]. Our results showed that laminin α5_CD239_spectrin staining was negative in activated SCs. Therefore, SCs are unlikely to modulate laminin α5_CD239_spectrin expression in new myofibers. Laminin α5_CD239_spectrin staining was positive in all embryonic myofibers labeled with eMHC. In contrast, CD239 was expressed in not only eMHC-positive myotubes, but also in the negative cells of regenerating skeletal muscles, suggesting that regeneration-specific signaling pathways modulate the expression of laminin α5_CD239_spectrin. As shown in the Results, the expression of CD239 was induced on day 4 and was maintained on day 7 after CTX-induced injury. Laminin α5_CD239_Spectrin positive-myofibers were with centrally located nuclei that represent regeneration. On the other hand, we also observed laminin α5_CD239_spectrin positive-myofibers in the soleus and diaphragm without centrally located nuclei. In addition, laminin α5-CD239-spectrin appears during the embryonic muscle maturation stage. Together, the expression of CD239-mediated linkage seems to be due to the common mechanical stress included in regenerative and maintenance process of myofibers.

When CD239-mediated linkage appeared in regenerating myotubes, muscular dystrophy was already severe. To rescue muscular dystrophy with CD239-mediated linkage, the expression of linkage is required before the degeneration of myotubes. *In silico* analysis using a dataset of mdx mice treated with prednisone showed that steroids induced the expression of laminin α5, CD239, and αII/βII-spectrin genes in skeletal muscles. Although triply immunostaining of the components is further required to define the formation of linkage in vivo, CD239-mediated linkage should be a complex associated with beneficial effects of steroid use in muscular dystrophy. To verify that CD239-mediated linkage rescues muscular dystrophy, we will examine the effects of prednisone in CD239 KO mice on mdx background.

Our findings revealed that laminin α5_CD239_spectrin may act as a compensatory complex in the sarcolemma to link the BM and cytoskeleton. However, the strength of CD239-mediated linkage remains unclear. Future studies will clarify the mechanism by which this CD239-mediated linkage can rescue myofiber disruption in muscular dystrophy. Finally, our results suggest that the CD239-mediated linkage may be a suitable molecular target for drug repositioning in muscular disorders, including sarcopenia.

## Methods

### Antibodies and regents

All primary antibodies used in this study are listed in Supplemental Table 1. CTX was purchased from Latoxan (Valence, France).

**Table 1:** Primary antibodies.

Antibody to	Epitope	Clone/ab no.	Host/Antigen Species	subclass	Source/Reference
Laminin α1	LN domain	–	Rabbit/Mouse	–	[38]
Laminin α2	N-terminus	4H8-2	Rat/Mouse	–	Merck, Kenilworth, NJ
Laminin α2	LG domain	–	Rbbit/Human	–	[39]
Laminin α3	LEc domain	–	Rabbit/Mouse	–	[40]
Laminin α4	LEc domain	–	Rabbit/Mouse	–	[41]
Laminin α5	LEb and L4b domains	8948	Rabbit/Mouse	–	[16]
Nidogen-1	–	ELM1	Rat/Mouse	IgG_2a_	Merck, Kenilworth, NJ
CD239	Extracellular domain	10–5	Rat/Mouse	IgG_2a_	[34]
CD239	Intracelullar domain	cyLu	Rabbit/Mouse	–	[19]
Dystroglycan	α-dystroglycan	3D7	Rat/Mouse	IgG_2a_	[24]
βII-spectrin	C-terminus	ab 72239	Rabbit/Human	–	Abcam, Cambridge, MA
Dystrophin	C-terminus	ab 15277	Rabbit/Human	–	Abcam, Cambridge, MA
Pired box protein Pax7	C-terminus	Pax7	Mouse/Human	IgG_1_	DSHB, Iowa City, IA
Myosin heavy chain 3	–	F1.652	Mouse/Human	IgG_1_	DSHB, Iowa City, IA

### Animals

C57BL/10ScSn-*Dmd*^mdx^/J mice (mdx mice) carrying a nonsense mutation in exon 23 of the dystrophin gene were purchased from Japan SLC, Inc. (Shizuoka, Japan). *Large*^myd^ mice were obtained from Jackson Laboratory (Bar Harbor, ME, USA). Myofiber-selective *fukutin*-cKO mice were generated in our previous study [23]. To obtain *fukutin*-cKO mice, flox *fukutin* mice (*fukutin*^lox/lox^) were crossed with muscle creatine kinase (MCK)-Cre mice expressing the Cre gene using the MCK promoter. The MCK promoter is active in differentiating muscle cells, and MCK expression remains constant throughout life. The mice were maintained in accordance with the animal care guidelines of Unitech Co. ltd., Kobe University, and Tokyo University School of Pharmacy and Life Science.

### Experimental muscle injury

Male C57BL/6 mice, aged 8–12 weeks, were purchased from Japan SLC (Shizuoka, Japan). CTX (30 μM; purified from the venom of the snake *Naja nigricollis*) was injected intramuscularly (30 μL into the tibialis anterior and 70 μL into the calf muscles). Saline solution was used as a mock injection. Samples were obtained at 0, 1, 3, 4, and 7 d after injection. The mice were sacrificed under deep anesthesia with thiopental.

### Immunohistochemistry

Mouse tissues were frozen in the Tissue-Tek optimum cutting temperature compound (Sakura Finetek, Tokyo, Japan). Sections (7 and 14 μm) were cut using a cryostat and air-dried. After blocking with 10 % normal goat serum, the sections were incubated with primary antibodies. The bound rabbit and rat IgGs were detected using secondary antibodies conjugated with Alexa Fluor 594 and Alexa Fluor 647, respectively (Thermo Fisher Scientific). The signals of Alexa Fluor 647 are converted to green in the figure. After washing with phosphate-buffered saline (PBS) (−), the sections were mounted in 90 % glycerol containing 0.1 × PBS and 1 mg/mL of *p*-phenylenediamine. Images were captured using BZ-X810 microscope (Keyence). The fluorescence intensity of the tissue was quantified using BZ-X800 analyzer (Keyence, Osaka, Japan). For SC staining, tissue sections were fixed with 4 % paraformaldehyde in PBS (−) and quenched with 0.1 M glycine in PBS (-). After washing with PBS (-), the sections were premetallized with PBS (−) containing 1 % Triton X 100 and blocked with 10 % normal goat serum containing the Fab fragment goat anti-mouse IgG (H + L) antibody. Mouse anti-Pax7 monoclonal antibody was detected using a subclass-specific secondary antibody conjugated with Alexa Fluor 647 (Thermo Fisher Scientific). The sections were visualized as described above. For triple immunostaining, anti-laminin α5 and βII-spectrin antibodies were labeled with Alexa488 and 594, respectively, using Zenon Rabbit IgG Labeling Kit (ThemoFisher Scientific). The signals of Alexa Fluor 488 are converted to light blue in the figure. After the triple immunostaining, images were captured within 3 h.

### Quantitative and statistical analyses

For quantitative evaluation of fluorescence intensity, at least 30 fibers were selected from the regenerative myofibers with centrally located nuclei in the gastrocnemius muscles. Immunofluorescence signals in the surrounding regions of myofibers were quantitatively measured using BZ-X800 analyzer (Keyence, Osaka, Japan). The medians obtained from each experiment were used as box-and-whisker plots. Statistical significance was determined using Welch’s *t* test. Comparisons between groups were performed using one-way analysis of variance with Dunnett’s post-hoc test. Statistical analyses were performed using R Studio v.1.4.1717. Significance was shown as follows: *p < 0.05.

### *In silico* analysis and visualization of single cell RNA-sequencing data on steroid-treated murine dystrophic muscles

Analysis and visualization of transcriptomic data were done using R v.4.1.2, assisted by RStudio v.1.4.1717 and the following R packages: dplyr v.1.0.7, and gplots v.3.1.1. RNA-seq data (GSE95682) of steroid-treated murine dystrophic muscles were downloaded from the GEO database. Genes related to muscle fiber cell adhesion were extracted from the normalized count data. The processed data were input into a heatmap.

### Study approval

Animal studies on mdx mice were approved by the Tokyo University School of Pharmacy and Life Science Committee on the Care and Use of Laboratory Animals (P18-68). All animal experiments were approved by the Animal Care and Use Committee of the Kobe University Graduate School of Medicine (P150605, P180901, and P200409) and Ehime University Graduate School of Medicine (05-O-70-1).

### CRediT authorship contribution statement

**Yamato Kikkawa:** Conceptualization, Investigation, Writing – original draft, Project administration, Funding acquisition. **Masumi Matsunuma:** Investigation, Validation. **Ryuji Kan:** Investigation. **Yuji Yamada:** Investigation. **Keisuke Hamada:** Investigation. **Motoyoshi Nomizu:** Investigation. **Yoichi Negishi:** Resources. **Shushi Nagamori:** Resources. **Tatsushi Toda:** Supervision, Resources, Writing – review & editing. **Minoru Tanaka:** Resources, Writing – review & editing. **Motoi Kanagawa:** Conceptualization, Investigation, Writing – review & editing.

## Declaration of Competing Interest

The authors declare that they have no known competing financial interests or personal relationships that could have appeared to influence the work reported in this paper.
