# Supplementary material for: Laminin α5_CD239_Spectrin is a candidate association that compensates the linkage between the basement membrane and cytoskeleton in skeletal muscle fibers
Source: Matrix Biol Plus. 2022 Aug 6;15:100118. doi: 10.1016/j.mbplus.2022.100118 (PMC9382564; doi:10.1016/j.mbplus.2022.100118)
Supplement: Supplementary data 1 [file mmc1.pdf]

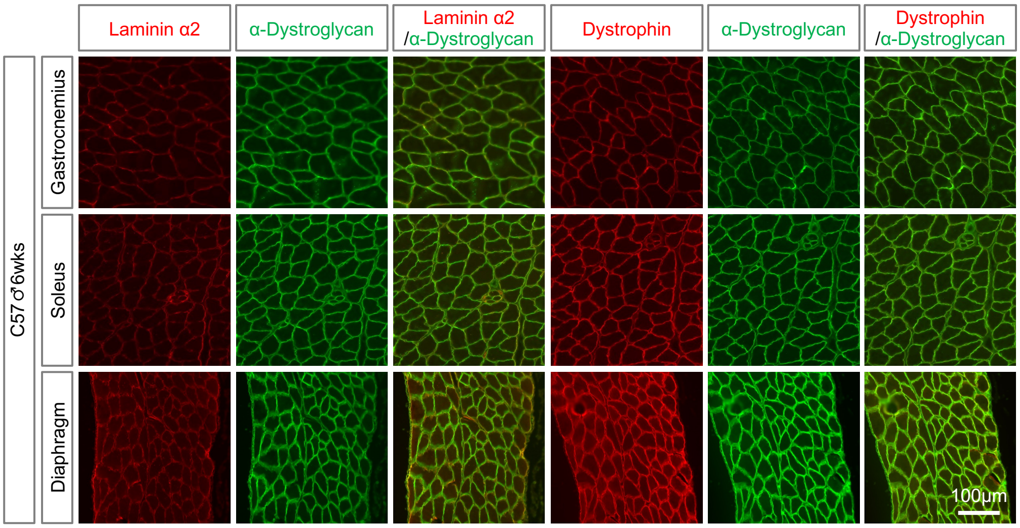

Figure S1. Expression levels of laminin α2, α-dystroglycan, and dystrophin in adult mouse skeletal muscles. Gastrocnemius (upper panel), soleus (middle panel), and diaphragm (lower panel) of 6-week-old male mouse. Frozen tissue sections were stained with antibodies against laminin α2, α-dystroglycan, and dystrophin, as indicated in panels. Bar: 100 μm.

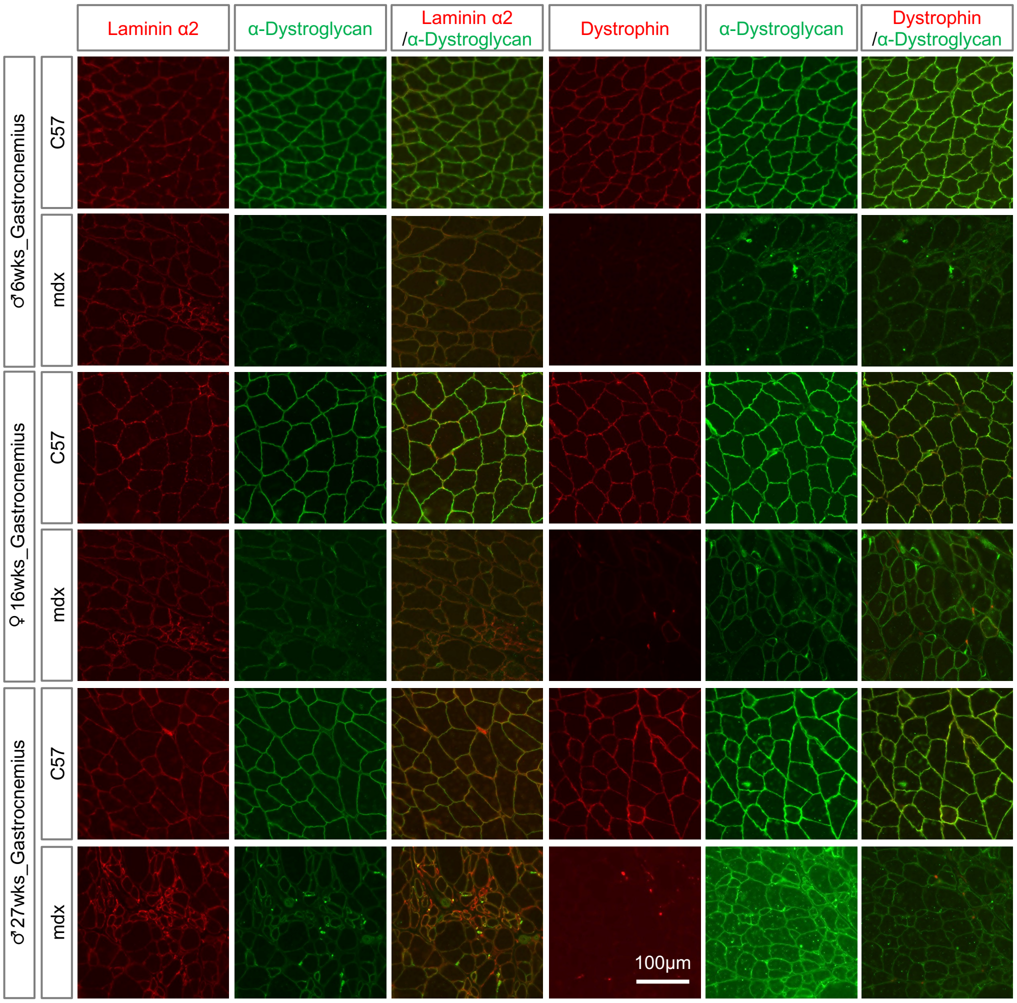

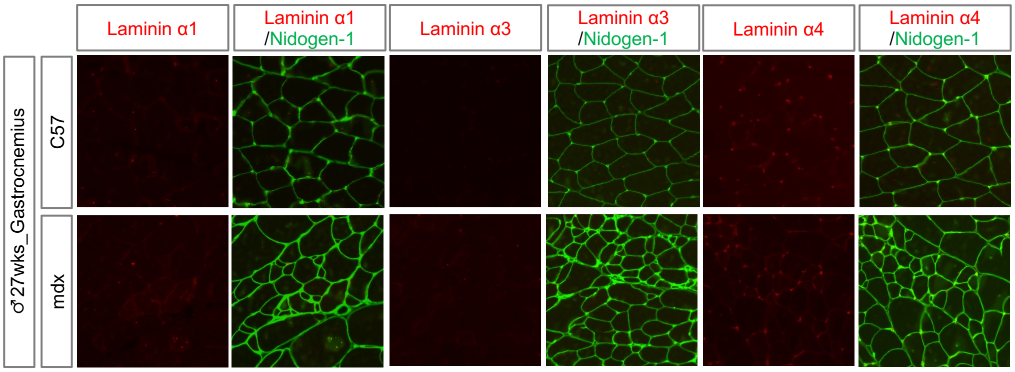

Figure S2. Expression levels of α-dystroglycan-mediated linkage and the other laminin α chains in skeletal muscles of Duchenne muscular dystrophy (DMD) model mice.

Gastrocnemius tissue sections of the control and mdx (6- and 27-week-old male and 16-week-old female) mice were stained with antibodies against α-dystroglycan, dystrophin, laminin α1, α2, α3, α4, and nidogen-1, as indicated in the panels. Bar: 100 μm.

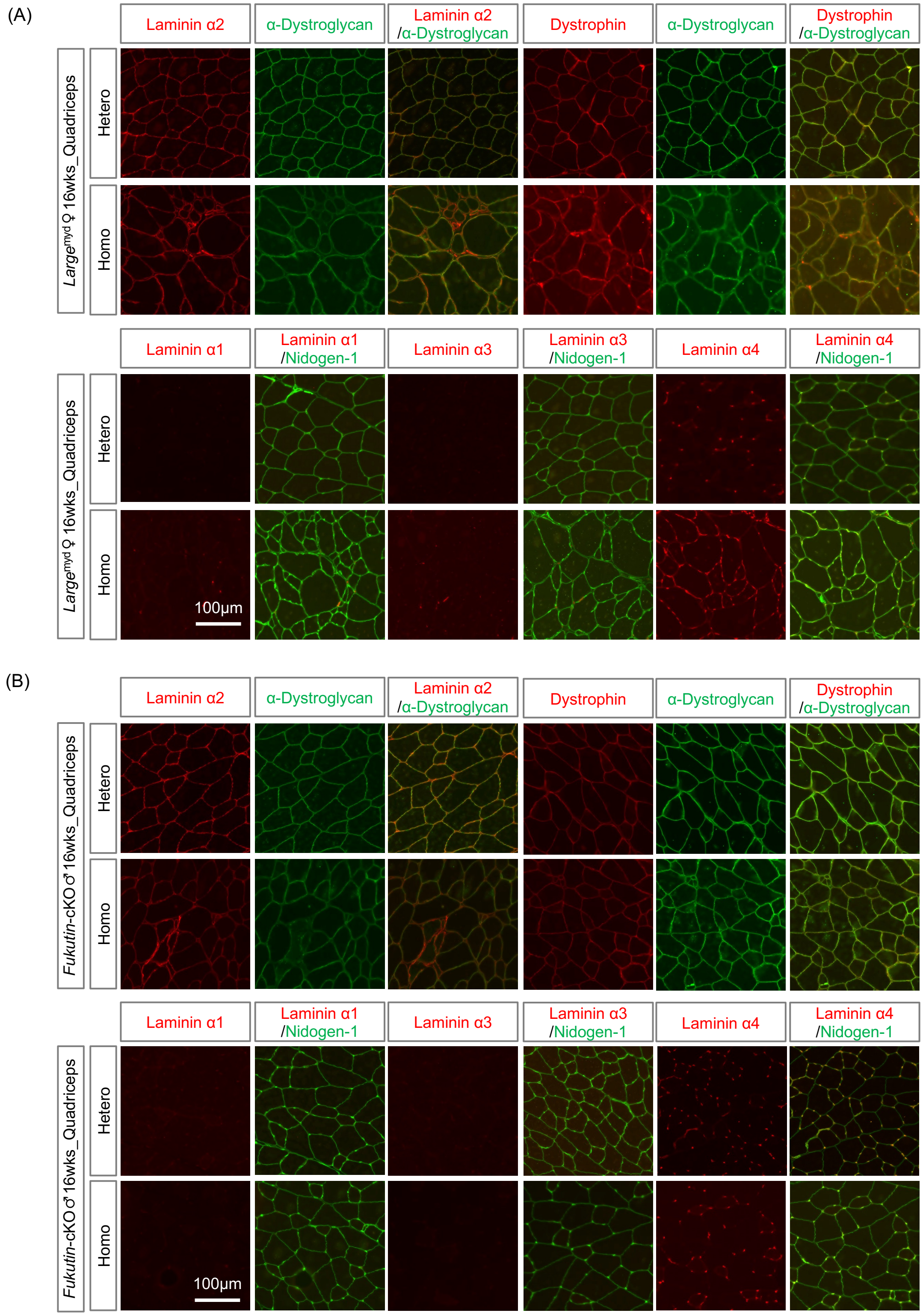

Figure S3. Expression levels of α-dystroglycan-mediated linkage and the other laminin α chains in skeletal muscles of congenital muscular dystrophy (CMD) model mice.

(A) Quadriceps muscles of heterozygous and homozygous *Large*$^{myd/myd}$ mice (16-week-old female). The tissue sections were stained with series of antibodies indicated in the panels. (B) Quadriceps muscles of heterozygous and homozygous MCK-*Fukutin*-cKO mice (16-week-old female). Bar: 100 μm.

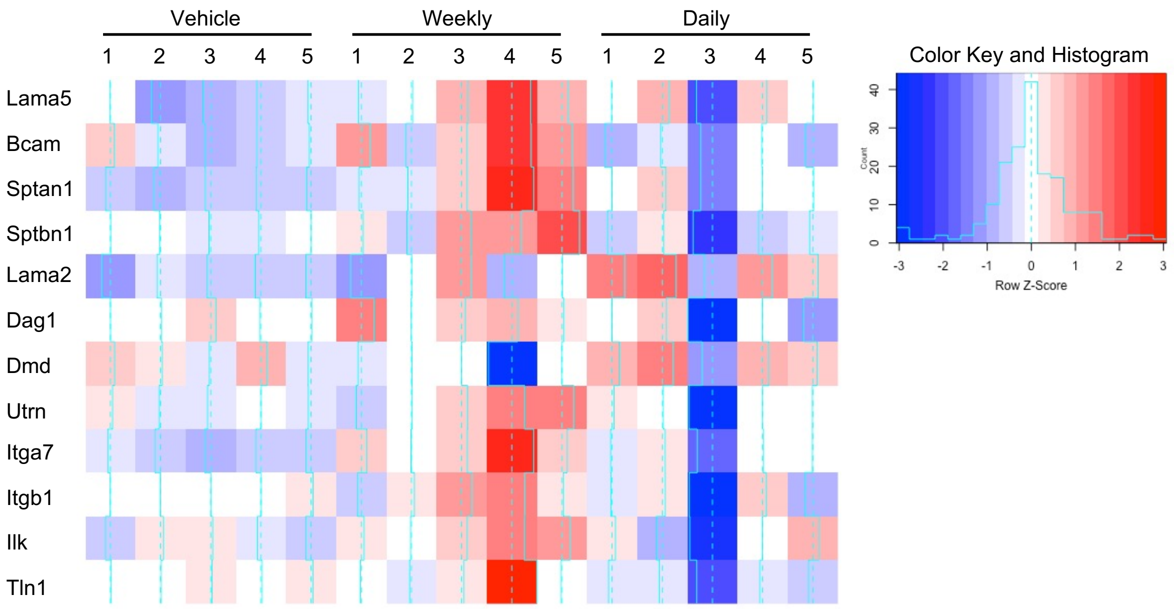

Figure S4. *In silico* analysis of steroid-treated murine dystrophic muscles.

Expression heatmap of genes related to the linkage between the basement membrane (BM) and cytoskeleton in the skeletal muscle of steroid-treated mdx mice. The RNA-seq data set was obtained from the Gene Expression Omnibus (GEO) database (GSE95682). The transcriptional profile was prepared from the quadriceps muscles of 6-month-old mdx mice treated without (vehicle) and with prednisone (weekly and daily) (n = 5 mice/group). Steroids were administered to mdx mice daily or weekly for four weeks. The color scale of the heatmap shows the abundance of each gene according to the Z-score. Solid lines represent Z-scores, and dotted lines indicate zero Z-scores in the heatmap. Lama5, laminin subunit α5; Bcam, basal cell adhesion molecule (CD239); Sptan1, αII-spectrin; Sptbn1, βII-spectrin; Lama2, laminin subunit α2; Dag1, dystroglycan; Dmd, dystrophin; Itga7, integrin α7; Itgb1, integrin β1; Ilk, integrin-linked protein kinase; Tln1, talin1.
